# Maternal immune activation induces sex-dependent behavioral differences in a rat model of schizophrenia

**DOI:** 10.3389/fpsyt.2024.1375999

**Published:** 2024-04-10

**Authors:** Yunxia Liu, Xiaoyi Hang, Yijie Zhang, Yilin Fang, Shanfang Yuan, Yi Zhang, Bin Wu, Yan Kong, Zihe Kuang, Wenjun Sun

**Affiliations:** ^1^ The Third Clinical Medical College, Beijing University of Chinese Medicine, Beijing, China; ^2^ College of Traditional Chinese Medicine, Beijing University of Chinese Medicine, Beijing, China; ^3^ Department of Encephalopathy, Beijing University of Chinese Medicine Third Affiliated Hospital, Beijing, China

**Keywords:** maternal immune activation, schizophrenia, Poly (I: C), sex difference, heterogeneousness

## Abstract

**Background:**

Maternal immune activation (MIA) is a mature means to construct a schizophrenia model. However, some preclinical studies have reported that a MIA-induced schizophrenia model seemed to have gender heterogeneity in behavioral phenotype. On the other hand, the MIA’s paradigms were diverse in different studies, and many details could affect the effect of MIA. To some extent, it is not credible and scientific to directly compare the gender differences of different MIA programs. Therefore, it is necessary to study whether the sex of the exposed offspring leads to behavioral differences on the premise of maintaining a consistent MIA mode.

**Methods:**

An animal model of schizophrenia was established by the administration of 10 mg/kg Poly (I: C) when dams were on day 9 of gestation. Then, a number of female and male offspring completed a series of behavioral tests during postnatal days 61–75.

**Results:**

Compared with the female control group (*n* = 14), female MIA offspring (*n* = 12) showed a longer movement distance (*d* = 1.07, *p* < 0.05) and higher average speed (*d* = 1.08, *p* < 0.05) in the open field test (OFT). In the Y maze test, the percentage of entering the novel arm of female MIA offspring was lower (*d* = 0.92, *p* < 0.05). Compared with the male control group (*n* = 14), male MIA offspring (*n* = 13) displayed less movement distance (*d* = 0.93, *p* < 0.05) and a lower average speed (*d* = 0.94, *p* < 0.05) in the OFT. In the Y maze test, the proportion of exploration time in the novel arm of male MIA offspring was lower (*d* = 0.96, *p* < 0.05). In the EPM, male MIA offspring showed less time (*d* = 0.85, *p* < 0.05) and a lower percentage of time spent in the open arms (*d* = 0.85, *p* < 0.05). Male MIA offspring also had a lower PPI index (76 dB + 120 dB, *d* = 0.81, *p* < 0.05; 80 dB + 120 dB, *d* = 1.45, *p* < 0.01).

**Conclusions:**

Our results showed that the behavioral phenotypes induced by prenatal immune activation were highly dependent on the sex of the offspring.

## 1 Introduction

A great deal of epidemiological evidence links maternal infection during pregnancy with several neuropsychiatric disorders including schizophrenia (SZ) and autism spectrum disorder (ASD) (1–3).

Research suggests that viral or bacterial infections during pregnancy act as initiators and mediate maternal immune activation (MIA) (4). MIA may directly affect maternal health through the abnormal release of inflammatory mediators (5). At the same time, the release of these inflammatory mediators may interfere with the development and differentiation of the fetal brain during pregnancy (6), potentially impacting on brain function and the behavior of offspring (7) and increasing the risk of diseases such as schizophrenia (8).

The development of MIA animal models is fundamental for the in-depth study of schizophrenia. So far, researchers have successfully established MIA models in rats, mice, rhesus monkeys, and more (9). The process of MIA is mainly realized by the administration of polyinsinic acid-polycytidylic acid [Poly (I: C)] or lipopolysaccharides (LPS) (10, 11). Poly (I: C) is a kind of RNA synthetic analog, while LPS is a kind of cell endotoxin. They are both powerful innate immune inducers acting on toll-like receptor (TLR) signal pathways, which respectively simulate the process of virus and bacteria infecting pregnant mothers (12). Based on these two classical MIA models, some studies have reported that the offspring exposed to a prenatal infection show a series of behavioral abnormalities similar to schizophrenia in adulthood, including cognitive dysfunction, sensory gating function impairment, anxiety-like behavior, social decline, and so on (13, 14). These studies provide substantial evidence of the causal relationship between MIA and schizophrenia-like behaviors of offspring.

However, current rodent studies on prenatal infection exposure have reported mixed and conflicting results. The majority of these studies showed that MIA does not induce the full spectrum of behavioral abnormalities (15, 16)—for example, in the study of Gray A and his colleagues (17), MIA-exposed rats only showed a decline in object recognition memory but did not exhibit other behavioral phenotypes such as impaired pre-impulse inhibition (PPI), loss of pleasure, and social decline. In another study, rats with prenatal Poly (I: C) exposure had social interaction defects, anxiety, and depression-like behaviors, while their performance in recognition memory was even better than that of the control group (14). The focus in the MIA field has started to turn to the diversity and heterogeneity of experimental outcomes in recent years. The researchers found that the disordered and sometimes contrary findings of MIA offspring were largely attributed to the experimental design (18). A series of interexperimental factors, including the vendors and batches of Poly (I: C) (19), dosage (20), route of injection, administration time (21), animal strain (22), and so on, have affected the outcome of prenatal immune activation.

Another potential influencing factor that should not be ignored is the sex of the offspring. Some clinical studies have reported that there are gender differences in the clinical symptoms of schizophrenia. Male schizophrenics seem to show more negative symptoms, while female schizophrenics tend to show more emotional symptoms (23). Whether there are similar gender differences in the animal model of schizophrenia mediated by maternal immune activation is still unknown. Although some researchers try to explore this problem in depth, these studies only studied certain aspects of schizophrenia, such as cognitive function (24). In addition, to avoid the possible interference of estrogen in the experiment, most researchers only focused on the influence of maternal immune activation on male offspring. Limited studies are focused on female offspring, but these studies reported inconsistent immune activation effects (24, 25). As described above, methodological differences and design details between studies may play a crucial role in MIA, which make it difficult to directly compare the gender differences in different research paradigms.

Studying the effect of prenatal Poly (I: C) exposure on offspring of different sexes is helpful to fully understand the influence of MIA on behavior phenotype and the gender differences of schizophrenia. On this basis, if researchers are interested in specific symptoms of schizophrenia (such as sensory gating impairment, anxiety, or hyperactivity), they can choose the sex of the animal to be tested according to the sex differences mediated by Poly (I: C) in these symptoms. This is of great significance for studying the specific symptoms of schizophrenia successfully and efficiently. In addition, a correct understanding of the gender differences in schizophrenia and its animal models can also guide the formulation of targeted prevention and treatment measures. Given the above-mentioned considerations, we carried out this study aiming at simultaneously exploring the effects of maternal Poly (I: C) exposure during pregnancy on schizophrenia-related behaviors of female and male offspring. We conducted a series of tests on spontaneous activity, anxious behavior, spatial working memory, intrinsic anhedonia, and sensory gating function to compare the behavior differences between female offspring and male offspring.

## 2 Materials and methods

### 2.1 Animals and treatment

**Figure 1:**
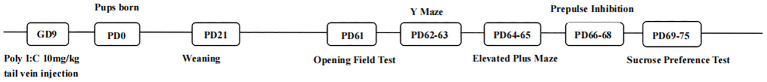
Timeline of the whole experiment.

A total of 10 pregnant female Wistar rats were obtained at gestation day (GD) 6 from Beijing Speifu Biotechnology Company, Ltd. These dams were of the same origin, age, and weight and were raised in the same housing environment. We targeted MIA to gestational day 9 (GD9), which was a critical period of embryonic development (14). The rats were divided randomly into two groups: (1) A part of the pregnant rats received a tail vein injection with polyinosinic: polycytidylic acid [Poly (I: C); Sigma-Aldrich] dissolved in 0.9% saline (10 mg/kg), and (2) the other part of pregnant rats were injected with an equivalent volume of saline. Parturition was considered postnatal day (PD) 0. Female and male pups were weaned on PD21. Each cage housed four to five from the same treatment group. The rats were housed in the specific pathogen free-grade animal laboratory of Beijing University of Chinese Medicine with environmentally controlled conditions (20°C–22°C, light/dark cycle: 8:00 am–8:00 pm/8:00 pm–8:00 am). Food and water were freely available. A series of behavioral tests was performed from PD61. The procedural timeline for the animal treatments is depicted in **Figure 1**. Other experimental details except gender were strictly controlled as much as possible. All experimental procedures were approved by the experimental animal ethics review committee of Beijing University of Chinese Medicine.

### 2.2 Sample size calculation

The sample size was calculated using G*Power (ver. 3.1.9.7, Heinrich-Heine-Universität Düsseldorf, Düsseldorf, Germany). According to the result of our preliminary experiment, the effect size was about 1.57. Using an alpha (α) level of 0.05 and a beta (β) level of 0.05, i.e., power = 95%, the estimated minimum sample size (*n*) for each group should be at least 12 samples. Considering that some rats may fail in the behavioral tests, we expanded the sample size with a 10% loss possibility. Thus, the actual sample size of each group was 14. Two female Poly (I: C) rats and one male Poly (I: C) rat failed to test in the prepulse inhibition test or the Y maze test. To avoid the potential impact of this failed test on their behaviors and ensure the consistency of test conditions for all rats, we did not retest the three rats. They were not included in the analysis of all behavioral tests.

### 2.3 Behavioral tests

A series of five behavioral tests was performed during PD61–75. Except for the sucrose water preference test, all behavioral experiments were performed between 9:00 am and 6:00 pm.

#### 2.3.1 Open field test (PD61)

The open field test was conducted to assess the locomotor activity and anxiety behavior of rats, following previously reported procedures (26). The experiment was carried out in a dark and quiet environment. A single rat was placed in the test box (88 cm × 88 cm × 45 cm) with a lamp covering the entire arena and allowed to explore the test box freely for 5 min. The behavior of the rat during the test was recorded and analyzed using the VisuTrack animal behavior analysis system (Shanghai Xinsoft Information Technology Co., Ltd.). The moving distance and time spent in the center and periphery zones were calculated.

#### 2.3.2 Y maze (PD62-63)

Spatial recognition memory was assessed by testing the rats’ ability to recognize previously explored sites and discover new places in the Y maze (27). The Y maze apparatus consisted of three black wooden arms (50 cm × 10 cm) radiating from a central triangle and separated by 120° from each other. The arms were named respectively as start arm, familiar arm, and novel arm. The test consisted of the training session and the test session. In the training session, a tested animal was put at the end of the start arm and allowed to explore only the start arm and the familiar arm for 5 min with the novel arm blocked by a partition. One hour after the training session, the novel arm was opened. The experiment entered the test session. The rats were still placed at the end of the start arm and allowed to explore all arms freely for 5 minutes. After testing, 75% alcohol was used to clean the floor of the maze, to reduce the interference of odor to the tested rats.

#### 2.3.3 Elevated plus maze test (PD64-65)

The elevated plus maze was a wooden structure elevated 50 cm above the floor used to test innate anxious behaviors (28). It consisted of two open arms (60 cm × 8 cm) and two closed arms (60 cm × 8 cm × 30 cm) radiating from a square center (8 cm × 8 cm). A single rat was placed on the central square of the elevated maze facing one of the closed arms. Then, the rat was allowed to explore freely for 5 min. Only when 60% of the whole rat body entered the closed arms was it considered to have entered the closed arms, and so was the criterion for the entry of open arms. The number of open arm and closed arm entries as well as the duration of time spent in the open arm and closed arm was scored. The rat was returned to the home cage after finishing the test. The percentage of open arm duration time was calculated using the formula ([time spent in the open arms]/[time spent in all arms] × rms%). The percentage of open arm frequencies was calculated using the formula ([open arm entries]/[total arm entries]  × ntr%). Both of these values can reflect the anxiety of animals. After the experiment on each rat, the four arms and the central square were carefully wiped with 75% ethanol, and the excrement of the rat was cleaned.

#### 2.3.4 Prepulse inhibition (PD66-68)

We used the paradigm of prepulse inhibition (PPI) to assess the rats’ sensorimotor gating ability (29). PPI was performed by using a single sound-attenuated chamber startle apparatus, and the results were analyzed by using a specific analysis software (Shanghai Xinsoft Information Technology Co., Ltd., China). At 1 day before the official PPI test, all rats were put into the experimental chamber with background white noise (68 dB, which could cover up the noise in the laboratory environment) for 15 min, which was necessary to adapt to the test equipment. The formal test began with a 5-min background noise adaptation period. Then, the rats received six startle trials (120-dB burst of sound stimuli lasting for 40 ms) to reduce the initial reaction of the animals to a platform level. Subsequently, the rats were given eight types of sound modes: (1) three separate prepulse stimulus of a 76-, 80-, or 84-dB burst of sound stimuli lasting for 20 ms; (2) a separate pulse stimulus of 120 dB lasting for 40 ms; (3) the combination of three kinds of prepulse stimulus and the startle reflex stimulation with white noise of 120 dB (with an interval of 100 ms), which was named prepulse + pulse; and (4) a no-stimulus mode with only background sound (68 dB). Each type of stimulus was presented randomly 10 times. All trials were separated by an average interval of 15 s. PPI percentage was calculated as (1 - average prepulse + pulse reaction amplitude/average pulse-alone reaction amplitude) * 100%.

#### 2.3.5 Sucrose preference test (PD69-75)

The sucrose preference test (SPT) was a reward-based behavioral test that was performed to reflect the intrinsic anhedonia state of animals (30). Throughout the experiment, the rats in each group were housed individually. For the first 3 days, the rats were trained to adapt to sucrose water by giving different drinking regimens. On the first day, the rats were given two bottles of 1% sucrose water for 24 h. On the second day, two bottles of sucrose solution were replaced with two bottles of pure water. Subsequently, the rats were continuously banned from food and water for 24 h. On the following day, the rats were given a bottle of pure water and a bottle of sucrose water at the same time. All water bottles are prelabeled and preweighed. Pure and sucrose water consumption was recorded within 12 h, and the rats were not disturbed. The sucrose water preference was calculated as follows: sucrose water preference (%) = sucrose water consumption (g)/total liquid (sugar water + pure water) consumption (g) × 100%.

### 2.4 Statistical analysis

The results from behavioral examinations were displayed as the means ± standard errors of the mean (SEMs). Statistical analysis of the data was carried out by IBM SPSS Software (ver. 23.0, Armonk, New York, USA). Comparisons of variables between groups [Female Ctrl vs. Female Poly (I: C) or Male Ctrl vs. Male Poly (I: C)] were analyzed using Student’s *t*-test. The *p*-value less than 0.05 was considered as the threshold of statistical significance. The effect sizes of the difference between groups were presented by Cohen’s *d* value (abbreviated as *d*). Graphical representations were generated by GraphPad Prism software (ver. 9.5.0, San Diego, CA, USA).

## 3 Results

### 3.1 Poly (I: C) exposure influenced the body weight of offspring in a sex-specific manner

**Figure 2:**
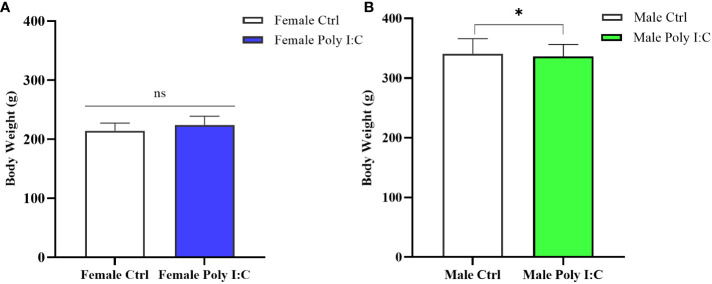
**I**mpact of Poly (I: C) treatment on the body weight of female and male offspring of dams. **(A)** Body weight of female offspring rats. **(B)** Body weight of male offspring rats. *n* = 19 in the Female Ctrl group, *n* = 36 in the Female Poly (I: C) group, *n* = 20 in the Male Ctrl group, *n* = 41 in the Male Poly (I: C) group. Female Ctrl, female offspring born to the control dams; Female Poly (I: C), female offspring born to the Poly (I: C)-treated dams; Male Ctrl, male offspring born to the control dams; Male Poly (I: C), male offspring born to the Poly (I: C)-treated dams. Poly (I: C) vs. saline: *p < 0.05 ; ns, not statistically significant.

On the day before the behavioral test (PD60), we measured the body weight of all rats. The result showed that there was a significantly higher body weight of the female MIA offspring compared to that of the female control offspring (**Figure 2A**, *d* = 0.72, *t* = -2.502, *p* < 0.05). However, after the treatment of dams with Poly (I: C), the body weight of male offspring was not significantly different from that of normal control rats (**Figure 2B**, *d* = 0.19, *t* = 0.745, *p > 0*.05).

### 3.2 Poly (I: C) exposure induced hyperactivity in female offspring and hypoactivity in male offspring

**Figure 3:**
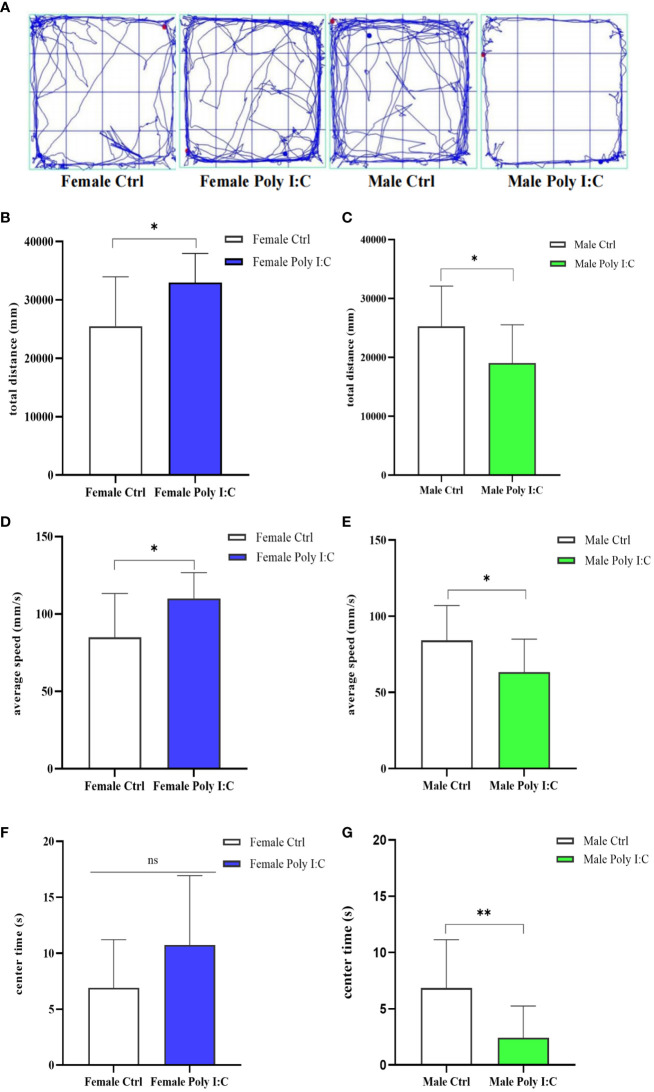
Impact of Poly (I: C) treatment on the spontaneous activity of female and male offspring of dams. **(A)** Representative movement trajectories of offspring born to dams receiving different treatments. **(B)** Total movement distance of female offspring. **(C)** Total movement distance of male offspring. **(D)** Movement velocity of female offspring. **(E)** Movement velocity of male offspring. **(F)** Duration in the central area of female offspring. **(G)** Duration in the central area of male offspring. *n* = 14 in the Female Ctrl and the Male Ctrl groups, *n* = 12 in the Female Poly (I: C) group, and *n* = 13 in the Male Poly (I: C) group. Female Ctrl, female offspring born to the control dams; Female Poly (I: C), female offspring born to the Poly (I: C) treated-dams; Male Ctrl, male offspring born to the control dams; Male Poly (I: C), male offspring born to the Poly (I: C)-treated dams. Poly (I: C) vs. saline: *p < 0.05; **p < 0.01; ns, not statistically significant.

We investigated the effect of prenatal Poly (I: C) therapy on the spontaneous activity of offspring rats. Representative movement trajectories of offspring are presented in **Figure 3A**. In female offspring, Poly (I: C) resulted in increased movement distance (**Figure 3B**, *d* = 1.07, *t* = -2.793, *p < 0*.05) and velocity of movement (**Figure 3D**, *d* = 1.08, *t* = -2.799, *p < 0*.05) during a 5-min open field test. Male offspring have the opposite performance. Because male Poly (I: C) rats in total travel distance (**Figure 3C**, *d* = 0.93, *t* = 2.424, *p < 0*.05) and travel velocity (**Figure 3E**, *d* = 0.94, *t* = 2.426, *p < 0*.05) were lower compared with male saline-treated offspring, the results seemed to indicate that prenatal Poly (I: C) administration induced hyperactivity in female rats and hypoactivity in male rats. Furthermore, we compared the duration of rats in the central area of the open field. Interestingly, Poly (I: C) decreased the time spent in the center zone in male offspring only (**Figure 3G**, *d* = 1.22, *t* = 3.146, *p < 0*.05). There were no significant differences between female Poly (I: C) and saline-treated offspring in center duration (**Figure 3F**, *d* = 0.72, *t* = -1.857, *p > 0*.05).

### 3.3 Poly (I: C) exposure induced spatial working memory defects in all offspring

**Figure 4:**
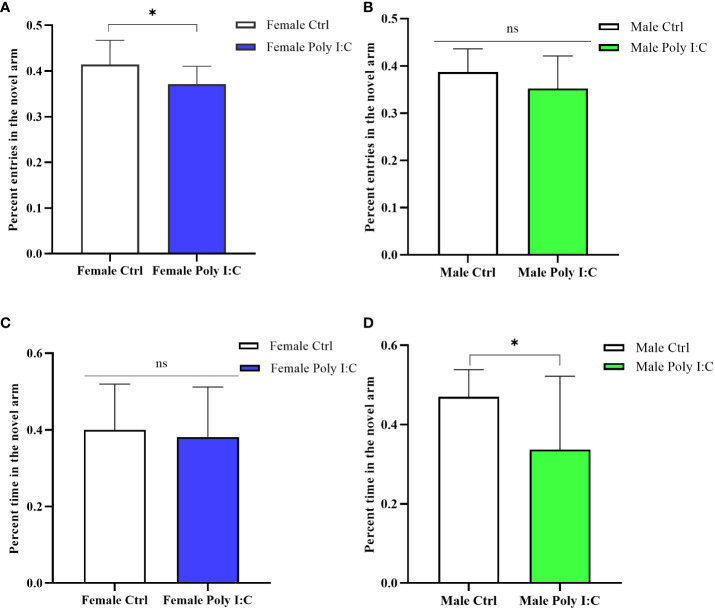
Impact of Poly (I: C) treatment on the spatial working memory activity of female and male offspring of dams. **(A)** Percentage novel arm frequencies of female offspring. **(B)** Percentage novel arm frequencies of male offspring. **(C)** Percentage novel arm duration of female offspring. **(D)** Percentage novel arm duration of male offspring. *n* = 14 in the Female Ctrl and the Male Ctrl groups, *n* = 12 in the Female Poly (I: C) group, *n* = 13 in the Male Poly (I: C) group. Female Ctrl, female offspring born to the control dams; Female Poly (I: C), female offspring born to the Poly (I: C)-treated dams; Male Ctrl, male offspring born to the control dams; Male Poly (I: C), male offspring born to the Poly (I: C)-treated dams. Poly (I: C) vs. saline: *p < 0.05; ns, not statistically significant.

In the Y maze testing session, female rats born to dams treated with Poly (I: C) were significantly less interested in exploring the novel arm. Because the percentage of entering the novel arm of female Poly (I: C) rats was lower than that of the controls (**Figure 4A**, *d* = 0.92, *t* = 2.316, *p* < 0.05). However, there was no significant difference in exploration time in the novel arm (**Figure 4C**, *d* = 0.15, *t* = 0.378, *p* > 0.05). Male offspring also showed defects of spatial working memory in the Y maze. The results displayed that although Poly (I: C) exposure did not affect the ratio of entering the novel arm of males (**Figure 4B**, *d* = 0.10, *t* = 1.524, *p* > 0.05), it significantly reduced the proportion of exploration time in the novel arm of male offspring (**Figure 4D**, *d* = 0.96, *t* = 2.451, *p* < 0.05). These results suggested that spatial working memory was affected by prenatal Poly (I: C) treatment.

### 3.4 Poly (I: C) exposure induced anxiety-like behavior in male offspring, but not in female offspring

**Figure 5:**
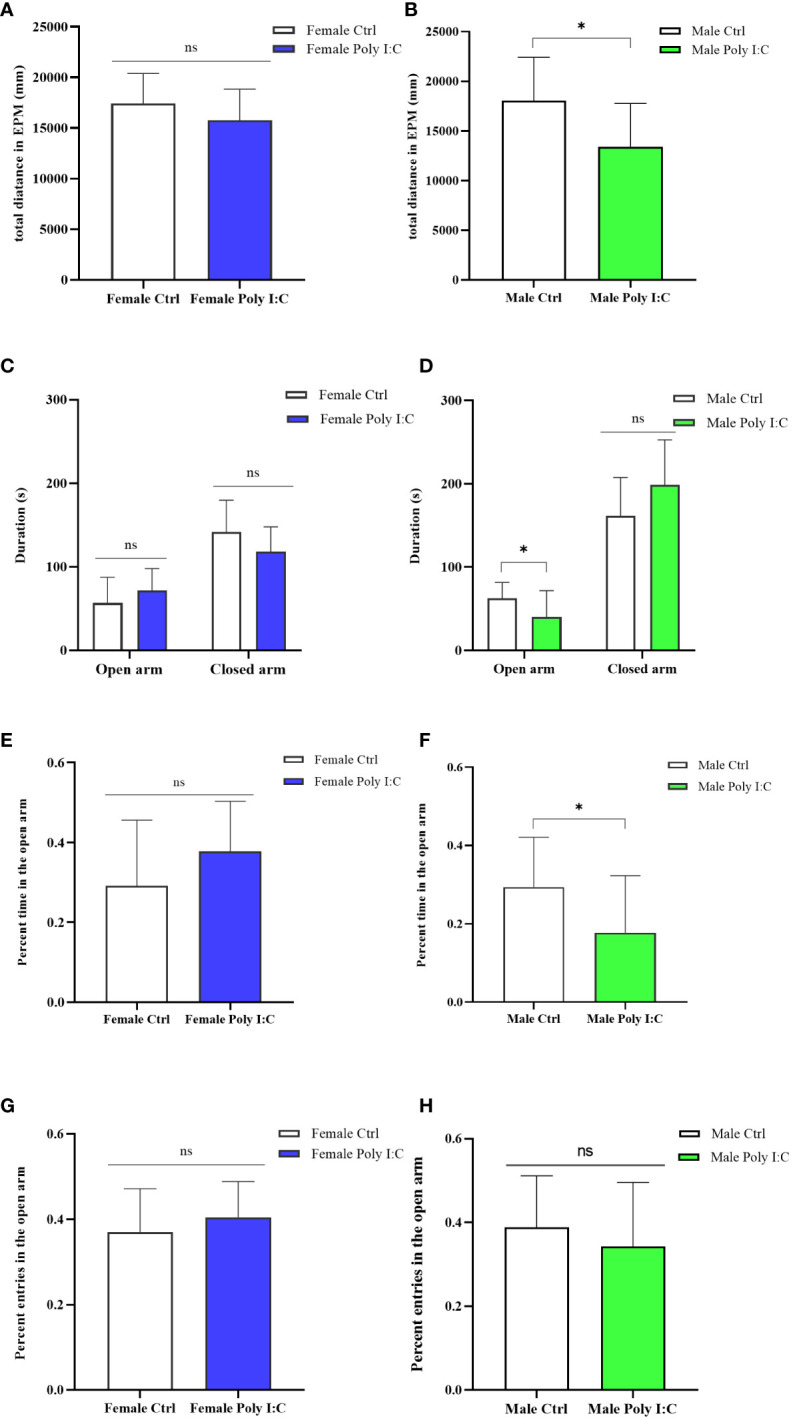
Impact of Poly (I: C) treatment on the anxiety-like behavior of female and male offspring of dams. **(A)** Total movement distance of female offspring in the elevated plus maze test. **(B)** Total movement distance of male offspring in the elevated plus maze test. **(C)** Duration spent in the open arm and the closed arm of female offspring. **(D)** Duration spent in the open arm and the closed arm of male offspring. **(E)** Percentage of open arm duration of female offspring. **(F)** Percentage of open arm duration of male offspring. **(G)** Percentage open arm frequencies of female offspring. **(H)** Percentage open arm frequencies of male offspring. *n* = 14 in the Female Ctrl and the Male Ctrl groups, *n* = 12 in the Female Poly (I: C) group, and *n* = 13 in the Male Poly (I: C) group. Female Ctrl, female offspring born to the control dams; Female Poly (I: C), female offspring born to the Poly (I: C)-treated dams; Male Ctrl, male offspring born to the control dams; Male Poly (I: C), male offspring born to the Poly (I: C)-treated dams. Poly (I: C) vs. saline: *p < 0.05; ns, not statistically significant.

We evaluated the anxiety-like behavior of rats by running an elevated plus maze test (EPM). Firstly, we evaluated the activity of the animals in the maze. The results showed that Poly (I: C) exposure of dams decreased the male offspring’s total distance in EPM (**Figure 5B**, *d* = 1.03, *t* = 2.7621, *p* < 0.05), while it did not affect the female offspring’s activity (**Figure 5A**, *d* = 0.55, *t* = 1.401, *p* > 0.05). The Poly (I: C) administration of pregnant rats did not alter the female offspring’s behavioral characteristics in the EPM apparatus, specifically the time spent in the open arms (**Figure 5C**, *d* = 0.52, *t* = -1.309, *p* > 0.05) and the closed arms (**Figure 5C**, *d* = 0.69, *t* = 1.731, *p* > 0.05), the percentage time spent in the open arms (**Figure 5E**, *d* = 0.59, *t* = -1.484, *p* > 0.05), and the percentage number of entries to the open arms (**Figure 5G**, *d* = 0.37, *t* = -0.942, *p* > 0.05). The behavior the of male offspring of Poly (I: C) dams in EPM was different from that of the female offspring. A significant decrease in time spent in the open arms (**Figure 5D**, *d* = 0.85, *t* = 2.189, *p* < 0.05) and the tendency to spend more time in the closed arms (**Figure 5D**, *d* = 0.74, *t* = -1.926, *p* > 0.05) were observed in the male Poly (I: C) group compared to the male control group. In addition, Poly (I: C) exposure to dams significantly decreased the male offspring’s percentage of time spent in the open arms (**Figure 5F**, *d* = 0.85, *t* = 2.214, *p* < 0.05). However, the closed arms entries in the male Poly (I: C) group were statistically insignificant compared with the male control group animals (**Figure 5H**, *d* = 0.33, *t* = 0.853, *p* > 0.05). These results indicated a sex-related restlessness due to antenatal Poly (I: C) treatment.

### 3.5 Poly (I: C) exposure induced the defect of sensory gating of male offspring, while female offspring were unaffected

**Figure 6:**
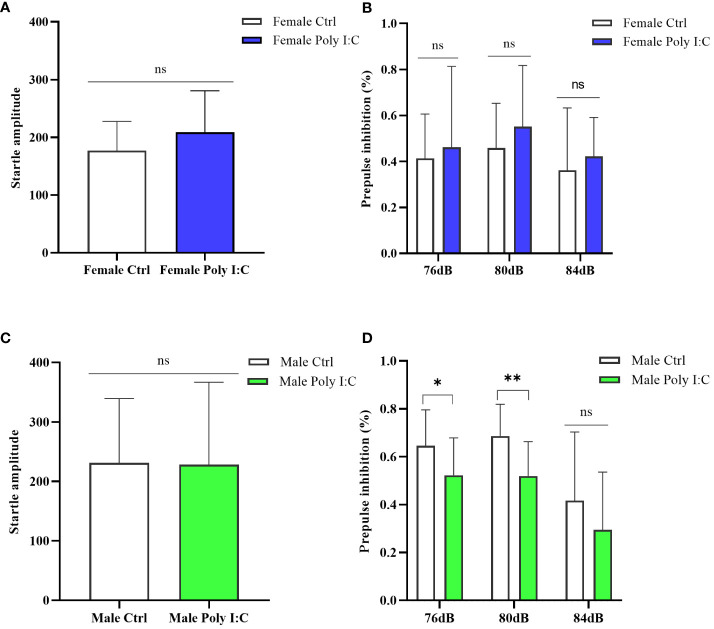
Impact of Poly (I: C) treatment on the sensory gating function of female and male offspring of dams. **(A)** Average startle amplitude of female offspring when the stimulus intensity was 120 dB. **(B)** Paradigm of prepulse inhibition (PPI) index of female offspring in different prepulse intensities. **(C)** Average startle amplitude of male offspring when stimulus intensity was 120 dB. **(D)** PPI index of male offspring in different prepulse intensities. *n* = 14 in the Female Ctrl and the Male Ctrl groups, *n* = 12 in the Female Poly (I: C) group, and *n* = 13 in the Male Poly (I: C) group. Female Ctrl, female offspring born to the control dams; Female Poly (I: C), female offspring born to the Poly (I: C)-treated dams; Male Ctrl, male offspring born to the control dams; Male Poly (I: C), male offspring born to the Poly (I: C)-treated dams. Poly (I: C) vs. saline: *p < 0.05; **p < 0.01; ns, not statistically significant.

The startle amplitude was not significantly affected by maternal Poly (I: C) exposure, regardless of the sex of the offspring (**Figure 6A**, Female Ctrl vs. Female Poly (I: C), *d* = 0.51, *t* = -1.324, *p* > 0.05; **Figure 6C**, Male Ctrl vs. Male Poly (I: C), *d* = 0.02, *t* = 0.064, *p* > 0.05). The sex-dependent PPI impairment induced by prenatal Poly (I: C) treatment was observed; thus, female offspring did not demonstrate any changes in PPI for all tested prepulse intensities (**Figure 6B**, 76 dB + 120 dB, *d* = 0.17, *t* = -0.439, *p* > 0.05; 80 dB + 120 dB, *d* = 0.40, *t* = -1.029, *p* > 0.05; 84 dB + 120 dB, *d* = 0.27, *t* = -0.673, *p* > 0.05), whereas male offspring displayed disturbed sensory gating for parts of the tested prepulse intensities (**Figure 6D**, 76 dB + 120 dB, *d* = 0.81, *t* = 2.093, *p* < 0.05; 80 dB + 120 dB, *d* = 1.45, *t* = 3.141, *p* < 0.01; 84 dB + 120 dB, *d* = 0.46, *t* = 1.195, *p* > 0.05).

### 3.6 Poly (I: C) exposure did not cause intrinsic anhedonia in offspring, whether female or male

**Figure 7:**
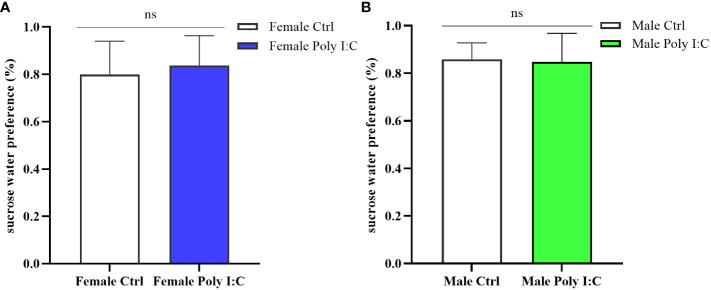
Impact of Poly (I: C) treatment on the sucrose water preference of female and male offspring of dams. **(A)** Proportion of sucrose water consumption of female offspring. **(B)** Proportion of sucrose water consumption of male offspring. *n* = 14 in the Female Ctrl and the Male Ctrl groups, *n* = 12 in the Female Poly (I: C) group, and *n* = 13 in the Male Poly (I: C) group. Female Ctrl, female offspring born to the control dams; Female Poly (I: C), female offspring born to the Poly (I: C)-treated dams; Male Ctrl, male offspring born to the control dams; Male Poly (I: C), male offspring born to the Poly (I: C)-treated dams. Poly (I: C) vs. saline: ns, not statistically significant.

We conducted a sucrose water preference test to explore whether the Poly (I: C) administration of pregnant rats would affect the depression-like behavior of offspring. The results showed that there was no difference in the percentage of preference for sucrose water between offspring born to rats treated with Poly (I: C) and those born to control rats. Neither the female offspring nor the male offspring showed significant intrinsic anhedonia (**Figure 7A**, Female Ctrl vs. Female Poly (I: C), *d* = 0.28, *t* = -0.722, *p* > 0.05; **Figure 7B**, Male Ctrl vs. Male Poly (I: C), *d* = 0.11, *t* = -0.292, *p* > 0.05), indicating that this effect was not affected by sex.

## 4 Discussion

We reported here that a single dose tail vein injection of 10 mg/kg Poly (I: C) at GD9 induced behavioral changes which were highly dependent on the sex of the offspring. For female offspring, maternal Poly (I: C) exposure led to higher body weight, excessive spontaneous activity as well as decreased cognitive memory. For male offspring, prenatal immune activation caused decreased spontaneous activity, spatial working memory deficit, anxiety-like behavior, and impaired sensorimotor gating function. Surprisingly, the behaviors of female offspring and male offspring were not affected by MIA in the sucrose water preference test.

Our present research displayed that the body weight of female offspring exposed to Poly (I: C) was higher than that of female offspring exposed to saline, while the weight of male MIA offspring was no different from that of the control males. The unusual change in body weight caused by MIA has been reported in previous animal studies. Chen et al. found that the prenatally exposed Poly (I: C) rats’ body weight was slightly increased, accompanied by a heavier liver and perirenal white adipose tissue (31). According to our research, female rats seemed to be more susceptible to prenatal Poly (I: C) exposure than male rats in terms of body weight. This effect may be related to the sex-specific distribution of adipose tissue and the regulation of sex hormones on body weight (32).

The total movement distance and average speed in the open field test reflected the spontaneous activity of the tested rats. So far, it was still inconclusive how MIA affected the spontaneous activity of offspring. This was because although there have been some studies on the correlation between prenatal immune activation and the offspring’s motor activity, it was difficult to reach a unanimous conclusion. Generally speaking, exposure to infection during pregnancy may increase or decrease the locomotor activity of offspring, and sometimes it even had no effect (14, 33, 34). Our research showed a gender-dependent spontaneous activity change. Concretely, maternal Poly (I: C) therapy mediated the hyperactivity of female offspring and the insufficiency of motor activity of male offspring. These results confirmed that sex was an important factor affecting the spontaneous activity phenotype of offspring when other experimental factors were uniformly set. Studies showed that sex steroid hormones played a role in the movement of humans and animals by affecting the dopamine system (35). Future research should thus investigate whether different levels of sex hormones may mediate differences in spontaneous activity between female and male offspring in the MIA group.

Anxiety is a common emotional symptom of schizophrenia (36). We used the open field test and the elevated plus maze test to evaluate the anxiety behavior of rats. Our results show that it was adult male offspring born to dams injected with 10 mg/kg poly (I: C), not female offspring, that presented elevated anxiety-like behaviors. A previous animal study that was designed similarly to ours reported that MIA resulted in the gradual deterioration of anxiety-related behaviors in male offspring from adolescence to adulthood (37). Taken together, a high-dose injection of Poly (I: C) in early pregnancy was effective in inducing the anxiety-like behavior of male offspring. However, this effect was not stable for female offspring. The sex differences in anxiety and other symptoms have been described to be related to the sexually dimorphic hypothalamus–pituitary–adrenals (HPA) axis. Nikolaos Kokras and others found that the anxiety level of male individuals was related to peripheral endogenous corticosterone, while the anxiety behavior of female individuals seemed to be less dependent on the influence of corticosterone (38).

Cognitive dysfunction is a core behavioral phenotype of schizophrenia, both for animal models of the disease and patients (39–41). Our present study showed that both female offspring and male offspring born to dams with Poly (I: C) exposure performed worse in the Y maze paradigm. Cognitive impairment has always been regarded as a consistent and stable feature of schizophrenia, which may occur at any stage of disease development (42, 43). The deficiency of cognitive ability would worsen with the increase of age (42). The rodent MIA model provided support for this view. Even though the research results may differ across the specific Poly (I: C) schemes in different studies, the aberrant cognitive behaviors of offspring seemed to be manifested at a certain stage of growth (44, 45). Thus, the existence of the behaviors related to cognitive dysfunction helped to confirm harmful factors from the previous maternal Poly (I: C) exposure that caused perturbed neurodevelopment in offspring.

Prepulse inhibition existed in the same manner in humans and rodents, which was a specific measure to evaluate schizophrenia. Prepulse inhibition deficit reflected the defect of information processing and the imbalance of inhibition ability in rats. The results showed that prenatal Poly (I: C) exposure mediated the decline of the PPI index in male offspring in early adulthood, while female offspring were not affected by MIA. This conclusion once again confirmed that there were gender differences in the final effect of immune activation in the schizophrenia model. The reasons why gender affected the offspring’s behaviors were still unknown. It may be attributed to the late onset of females (46) and the potential influence of sex hormones on pathological processes (47)—for example, a study of Gogos and colleagues found that estrogen and progesterone prevented rats from showing the disruption of prepulse inhibition of sound startle mediated by 5-hydroxytryptamine serotonin-1A [5- HT (1A)] receptor (48).

Anhedonia referred to the ability to experience happiness, which was considered to be related to a dysfunction of the reward system in the brain. To evaluate whether Poly (I: C) exposure would induce the offspring’s anhedonia, the sucrose water preference test was used in our study. The results showed that neither the female offspring nor the male offspring receiving maternal immune activation showed a significant decline in sugar water preference. Although anhedonia has long been regarded as one of the negative symptoms of schizophrenia, some studies have gradually found that anhedonia was not necessary for schizophrenia, and schizophrenia patients could have the intact ability to experience present pleasure (49). Previous studies on schizophrenia rat model also reported that MIA did not make the offspring display anhedonia in adulthood (50). One view was that Poly (I: C) exposure in late pregnancy was more likely to induce negative symptoms. One study successfully promoted the offspring’s expression of anhedonia in adulthood under this MIA mode (51). We noticed that the implementation time of the sugar water preference test in this study was at a later stage of adulthood compared with our study. We thought that the performance of anhedonia in behavioral tests may be related to the progress of the disease course.

Our work provided new evidence for sex heterogeneity in the behavioral phenotype of an animal model of schizophrenia. Specifically, in the MIA-induced schizophrenia model, there were significant differences in weight, spontaneous activity, anxiety behavior, and sensory gating function between male and female offspring. Considering that, in our study’s open field test the female MIA offspring exhibited a hyperactive behavior, whereas the male MIA offspring was demonstrated to have much less spontaneous activity, we speculated that the Poly (I: C) challenge of dams made the female offspring have a tendency to display positive symptoms and the male offspring tend to display negative symptoms, which was consistent with research on schizophrenia from a clinical perspective (52). The sex specificity of the behavior phenotypes associated with schizophrenia may be partially explained by the expression of estrogen receptor β (ERβ) as ERβ expression has been reported to show sex differences in developing brains (53). In support of this view, a study in a schizophrenia model found that prenatal exposure to Poly (I: C) (5 mg/kg) had an impact on the expression of ERβ in offspring (21). Therefore, prenatal immune activation may directly affect the expression of sex hormones and their related receptors and ultimately mediate sex-dependent schizophrenia phenotype. However, we did not measure these molecular indicators because of inadequate study conditions. This represented a constraint on our study. Further study to evaluate the role of sex hormones and their receptors in schizophrenia behavior phenotype deserves attention.

Whether MIA has a gender-specific impact on anhedonia is yet to be determined because our study only observed the impact of how prenatal immune activation affected the offspring’s behavioral phenotype in early adulthood. Clinical evidence shows that some schizophrenics are late onset, that is, some symptoms of schizophrenia first appear in middle adulthood (54). There may be a similar disease progression pattern in the rat model of schizophrenia induced by Poly (I: C). As a result, the symptoms of anhedonia in the offspring are probably not manifested in early adulthood. We believe that the sex differences of MIA offspring will be more significant in the middle or late adulthood of MIA offspring, e.g., those corresponding to menopause. However, limited by time and research conditions, we did not carry it out further in this study.

One possible mechanism of MIA mediating schizophrenia was that it could induce an inflammatory cascade. However, there may be variations in the inflammatory response induced by Poly (I: C) exposure in different pregnant rats. Furthermore, the tail vein injection may exert extra pressure on animals, triggering a slight immune reaction. Our study did not eliminate this effect by establishing the wild-type group. Our findings were limited to some extent by these situations. Therefore, it may be necessary to verify the immune inflammatory response scientifically to discuss the gender differences of MIA-induced schizophrenia rats.

## 5 Conclusions

Overall, our study confirmed that Poly (I: C) exposure mediated different behavioral phenotypes of schizophrenia between female offspring and male offspring in early adulthood. This provides new support for the view that the complex outcome effect of a schizophrenia model depends on the sex of the offspring because the destruction modes of neural development by different MIA schemes are different. Therefore, future studies need to verify the influence of offspring gender on behavior phenotypes and specific pathological changes in different immune attack schemes in more detail to better understand the internal mechanism of gender heterogeneity in schizophrenia animal models.

## Data availability statement

The original contributions presented in the study are included in the article/**Supplementary Material**. Further inquiries can be directed to the corresponding author.

## Ethics statement

The animal study was approved by the experimental animal ethics review committee of Beijing University of Chinese Medicine. The study was conducted in accordance with the local legislation and institutional requirements.

## Author contributions

YL: Conceptualization, Methodology, Writing – original draft. XH: Methodology, Writing – original draft. YJZ: Methodology, Writing – original draft. YF: Methodology, Writing – original draft. SY: Methodology, Writing – original draft. YZ: Methodology, Writing – original draft. BW: Software, Writing – original draft. YK: Software, Writing – original draft. ZK: Visualization, Writing – original draft. WS: Conceptualization, Funding acquisition, Supervision, Writing – review & editing.
