# Supplementary material for: Maternal immune activation induces sex-dependent behavioral differences in a rat model of schizophrenia
Source: Front Psychiatry. 2024 Apr 10;15:1375999. doi: 10.3389/fpsyt.2024.1375999 (PMC11040086; doi:10.3389/fpsyt.2024.1375999)
Supplement: Supplementary file 1 [file DataSheet_1.pdf]

Supplemental materials: the outcome difference between the whole MIA model group vs Control group

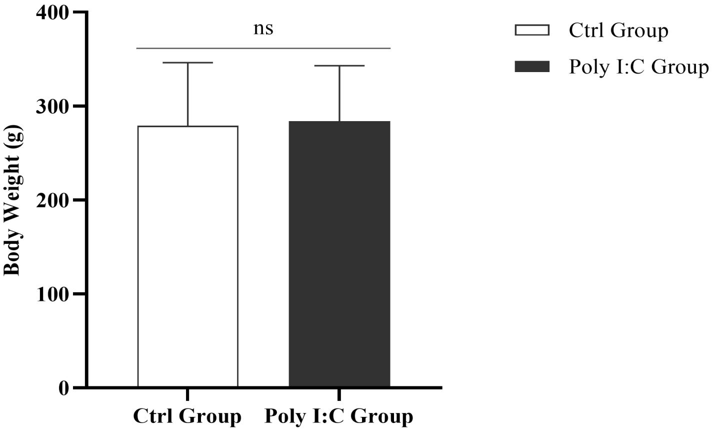

Figure S1. The impact of Poly I:C treatment on the body weight of the offspring of dams (d = 0.08, t = -0.396, $p > 0.05$)**.** (n = 39 in the Ctrl Group, n = 77 in the Poly I:C Group. Ctrl Group, refers to the offspring born to the control dams; Poly I:C Group, refers to the offspring born to the Poly I:C treated dams. Ctrl vs Poly I:C: ns, not significant statistically)

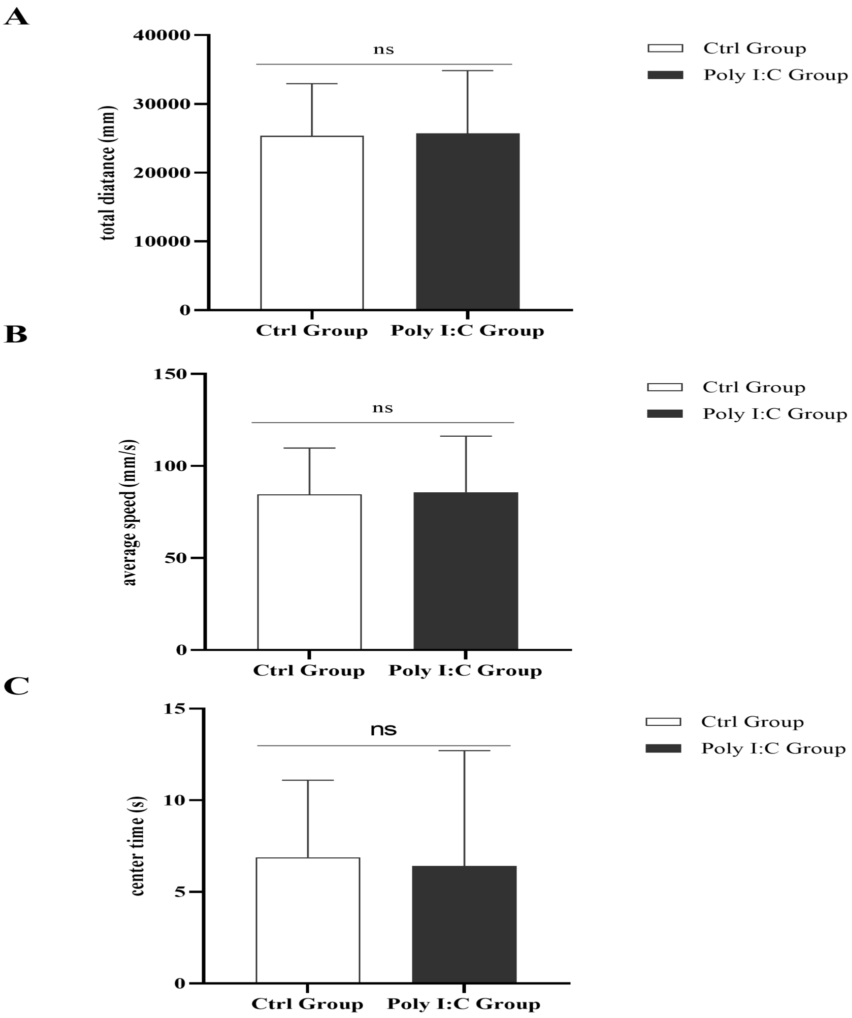

Figure S2. The impact of Poly I: C treatment on the spontaneous activity of the offspring of dams. (A)The total movement distance of the offspring (d = 0.04, t = -0.153, $p > 0.05$). (B) The movement velocity of the offspring (d = 0.04, t = -0.156, $p > 0.05$). (C) The duration in the central area of the offspring (d = 0.09, t = 0.313, $p > 0.05$). (n = 28 in the Ctrl Group, n = 25 in the Poly I: C Group. Ctrl Group, refers to the offspring born to the control dams; Poly I:C Group, refers to the offspring born to the Poly I: C treated dams. Poly I: C vs saline: ns, not significant statistically)

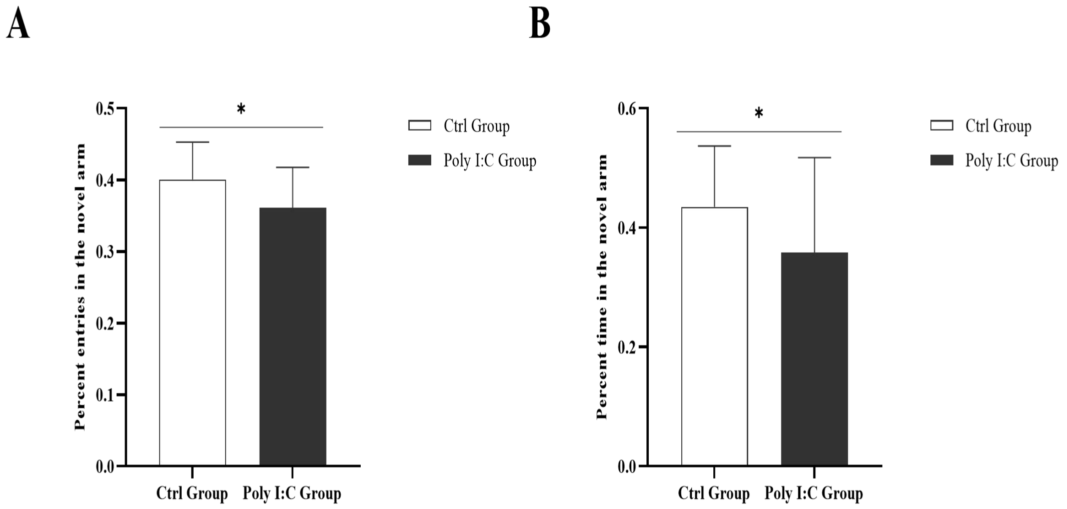

Figure S3. The impact of Poly I: C treatment on the spatial working memory activity of the offspring of dams. (A) The percentage novel arm frequencies of the offspring ($d = 0.73$, $t = 2.644$, $p < 0.05$). (B) The percentage novel arm duration of female offspring ($d = 0.57$, $t = 2.112$, $p < 0.05$). (n = 28 in the Ctrl Group, n = 25 in the Poly I:C Group. Ctrl Group, refers to the offspring born to the control dams; Poly I: C Group, refers to the offspring born to the Poly I: C treated dams. Poly I: C vs saline: *$p < 0.05$)

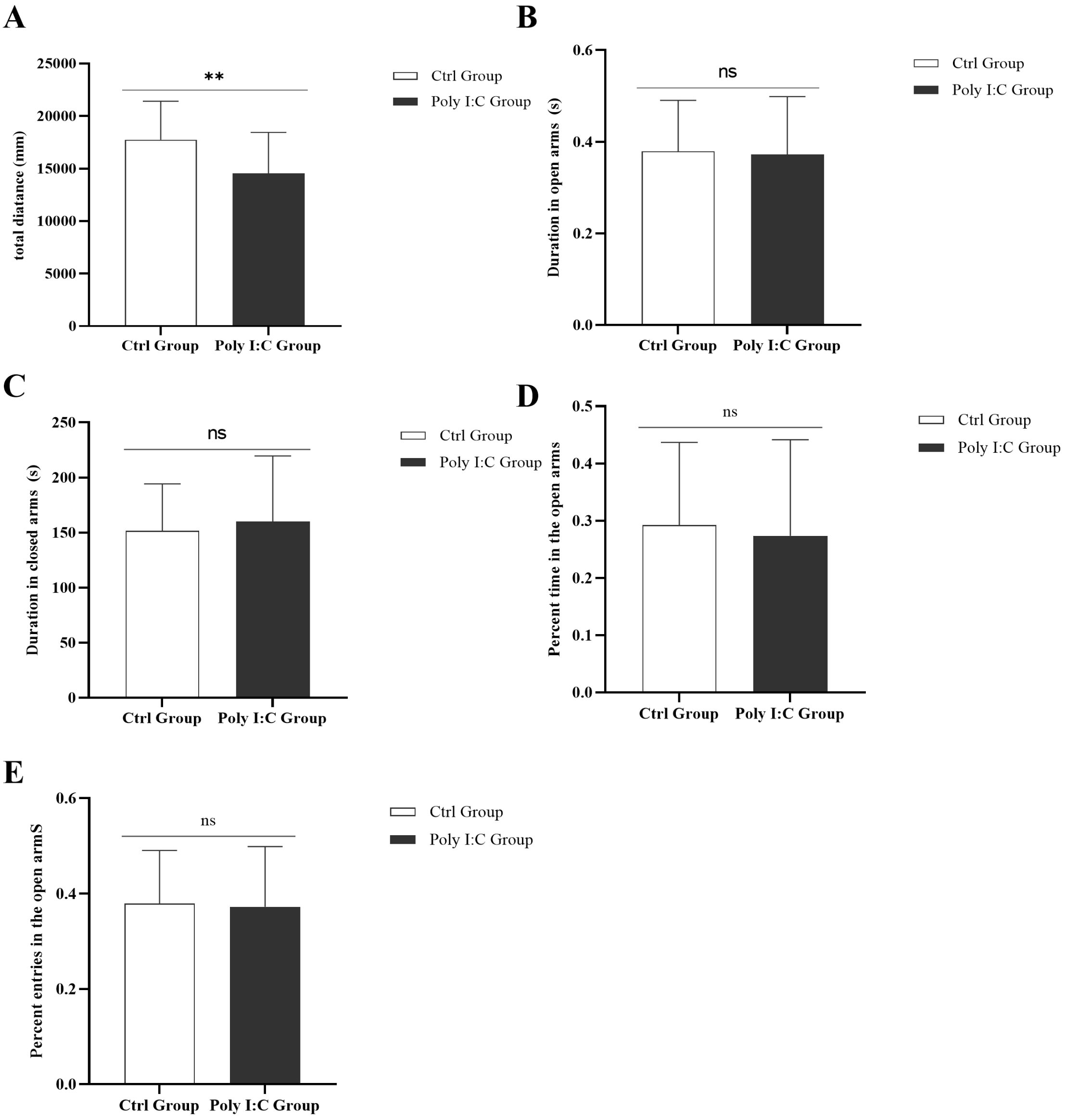

Figure S4. The impact of Poly I: C treatment on the anxiety-like behavior of the offspring of dams. (A) The total movement distance of the offspring in the elevated plus maze test (d = 3.07, t = -0.156, p ＜ 0.01). (B) The duration spent in the open arms of the offspring (d = 0.15, t = 0.553, p > 0.05). (C) The duration spent in the closed arms of the offspring (d = 0.16, t = -0.598, p > 0.05). (D) The percentage of open arms duration of the offspring (d = 0.12, t = 0.445, p > 0.05). (E) The percentage open arms frequencies of the offspring (d = 0.05, t = 0.201, p > 0.05). (n = 28 in the Ctrl Group, n = 25 in the Poly I:C Group. Ctrl Group, refers to the offspring born to the control dams; Poly I:C Group, refers to the offspring born to the Poly I:C treated dams. Poly I: C vs saline: **p < 0.01; ns, not significant statistically)

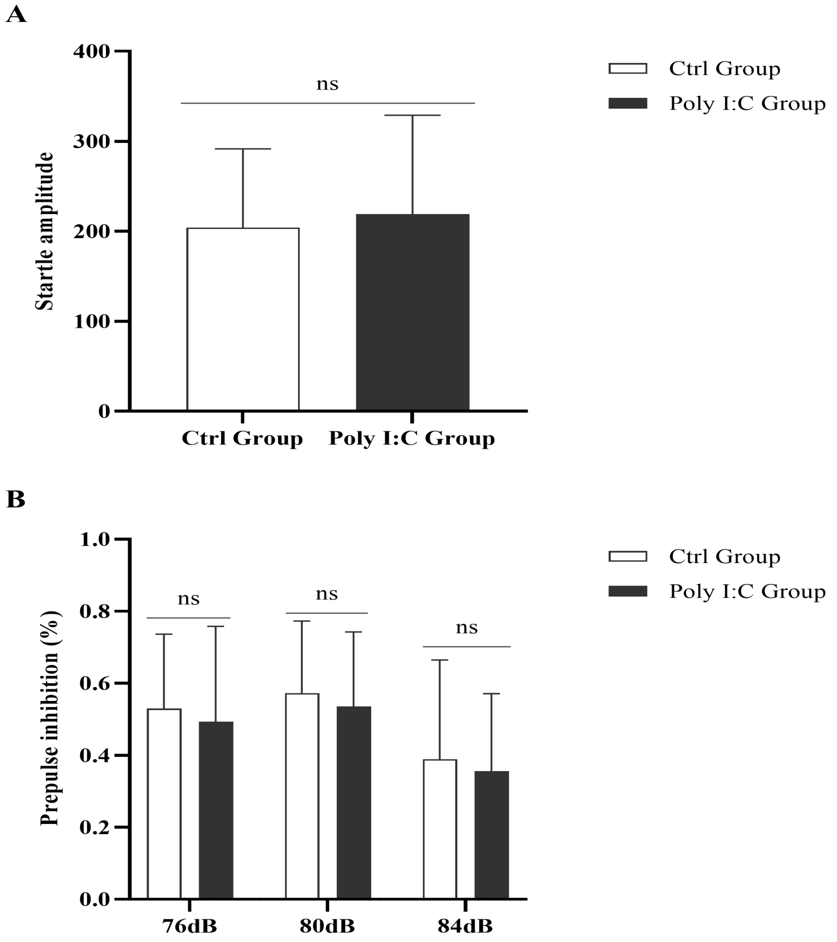

Figure S5. The impact of Poly I: C treatment on the sensory gating function of female and male offspring of dams. (A) The average startle amplitude of the offspring when stimulus intensity was 120 dB ($d = 0.15$, $t = -0.546$, $p > 0.05$). (B) PPI index of the offspring in different pre-pulse intensities (76 dB+120 dB, $d = 0.15$, $t = 0.563$, $p > 0.05$; 80 dB+120 dB, $d = 0.18$, $t = 0.671$, $p > 0.05$; 84 dB+120 dB, $d = 0.13$, $t = 0.484$, $p > 0.05$). (n = 28 in the Ctrl Group, n = 25 in the Poly I:C Group. Ctrl Group, refers to the offspring born to the control dams; Poly I:C Group, refers to the offspring born to the Poly I:C treated dams. Poly I: C vs saline: ns, not significant statistically)

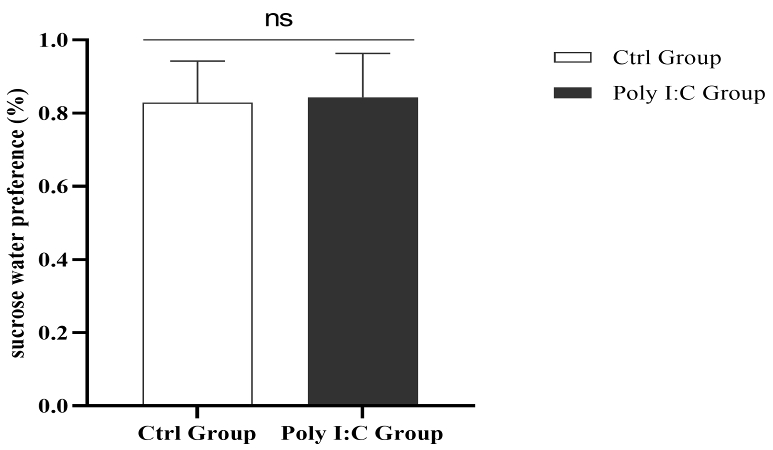

Figure S6. The impact of Poly I: C treatment on the proportion of sucrose water consumption of the offspring of dams (*d* = 0.12, *t* = -0.430, *p* > 0.05)**.** (n = 28 in the Ctrl Group, n = 25 in the Poly I:C Group. Ctrl Group, refers to the offspring born to the control dams; Poly I:C Group, refers to the offspring born to the Poly I:C treated dams. Poly I: C vs saline: ns, not significant statistically)
